# Review of patient-reported outcome measures in chronic hepatitis C

**DOI:** 10.1186/1477-7525-10-92

**Published:** 2012-08-07

**Authors:** Leah Kleinman, Sally Mannix, Yong Yuan, Shannon Kummer, Gilbert L’Italien, Dennis Revicki

**Affiliations:** 1 United BioSource Corporation, Bethesda, MD, USA; 2 Bristol-Myers Squibb Company, Global Health Outcomes, Plainsboro, NJ, USA; 3 Senior Research Scientist, United BioSource Corporation, 1417 4th Avenue, Suite 510, Seattle, WA, 98101, USA

**Keywords:** Hepatitis C, Patient-reported outcomes, Literature review, Health-related quality of life

## Abstract

**Background:**

Chronic hepatitis C (CHC) and its treatment are associated with a variety of patient-reported symptoms and impacts. Some CHC symptoms and impacts may be difficult to evaluate through objective clinical testing, and more easily measured through patient self-report. This literature review identified concepts raised by CHC patients related to symptoms, impacts, and treatment effects, and evaluated integration of these concepts within patient-reported outcome (PRO) measures. The goal of this work was to provide recommendations for incorporation of PRO measurement of concepts that are relevant to the CHC experience into CHC clinical trial design.

**Methods:**

A three-tiered literature search was conducted. This included searches on concepts of importance, PRO measures used in clinical trials, and existing PRO measures. The PRO Concept Search focused on reviewing issues raised by CHC patients about CHC symptoms, disease impact, and treatment effects. The CHC Trials with PRO Endpoints Search reviewed clinical trials with PRO endpoints to assess differences between treatments over time. The PRO Measure Search reviewed existing PRO measures associated with the concepts of interest.

**Results:**

This multi-tiered approach identified five key concepts of interest: depression/anxiety, fatigue, flu-like symptoms, cognitive function, insomnia. Comparing these five concepts of interest to the PRO measures in published CHC clinical trials showed that, while treatment of CHC may decrease health-related quality of life in a number of mental and physical domains, the PRO measures that were utilized in published clinical trials inadequately covered the concepts of interest. Further review of 18 existing PRO measures of the concepts of interest showed only four of the 18 were validated in CHC populations.

**Conclusions:**

This review identified several gaps in the literature regarding assessment of symptoms and outcomes reported as important by CHC patients. Further research is needed to ensure that CHC clinical trials evaluate concepts that are important to patients and include measures that have evidence supporting content validity, reliability, construct validity, and responsiveness.

## Background

Hepatitis C virus (HCV) is a worldwide public health concern that affects between 170 and 200 million people. Approximately 60% to 80% of patients with acute hepatitis C viral infection eventually develop chronic hepatitis C (CHC) [1,2]. Individuals with CHC may experience symptoms including nausea, fatigue, musculoskeletal and abdominal pain, and headaches [3]. Neuropsychiatric symptoms such as depression, fatigue, irritability, and malaise are reported by patients with both acute and chronic hepatitis infection, with depression being the most frequently reported [4]. These symptoms are bothersome to patients and often result in reduced health-related quality of life (HRQL) [5,6].

The current standard of care (SOC) for treating CHC patients with chronic infection is a regimen of interferon alpha (IFN-α) and ribavirin (RBV). This combination has been found to result in the highest sustained response rates in clinical trials [7-9]. Although treatments demonstrate acceptable efficacy, concerns about the severity of the side effects associated with treatment exist. High rates of treatment noncompliance and apprehension about starting treatment bolster these concerns [10]. Treatment with IFN-α and RBV can cause many severe symptoms including physical fatigue, flu-like symptoms, hair loss, gastrointestinal (GI) symptoms, headache, and neuropsychological symptoms including mental fatigue, concentration difficulties, depression, and irritability [1]. These symptoms in turn impact HRQL and patients’ ability to perform everyday activities [3].

CHC symptoms and treatment effects are difficult to evaluate through objective clinical testing, although they can be measured through patient self-report; that is, patient-reported outcome (PRO) measures. The use of a PRO measure provides insight into the patient perspective on the impact of disease and treatment [11]. PRO measures must have content validity, meaning there must be clear evidence demonstrating that the patients’ perspective has been taken into account during instrument development. Systematic development of PRO measures require qualitative research to identify key concepts from patients’ perspectives, clinician input, careful development of item content and response scales, cognitive debriefing interviews, and the evaluation of psychometric characteristics (i.e., reliability, validity, responsiveness) [12]. In addition, the guidance from the Food and Drug Administration (FDA) on the application of PRO instruments for product labeling recommends validation in the population of interest, including qualitative research to elicit key concepts, cognitive interviewing, and evaluation of psychometric properties [13].

## Objective

The purpose of this three-tiered literature review was to identify what concepts patients raise with regard to CHC symptoms, disease impact, and treatment effects, and to assess whether measurement of these concepts have been integrated into PRO measures and clinical trials. This was achieved through identification of concepts, identification and review of PRO measures that cover the key concepts that were identified, and review of published clinical trial data to determine what concepts are being measured in trials through PRO measures and how PRO endpoints reflect clinical changes.

## Methods

A three-tiered literature review was conducted. This included searches on concepts of importance to CHC patients, PRO measures used in clinical trials, and existing PRO measures. The CHC PRO Concept Search was designed to identify qualitative and quantitative research reporting on the CHC patient experience to determine concepts of importance to the CHC population. The CHC Trials with PRO Endpoints Search was designed to identify CHC clinical trials with PRO endpoints to determine whether PRO results show differentiation between treatments over time. The PRO Measure Search was designed to identify PRO measures that were either used within CHC populations or contained domains that mapped to the concepts of interest selected during the CHC PRO Concept Search.

### Inclusion and exclusion criteria

A priori inclusion and exclusion criteria were used to guide identification and selection of literature and PRO measures (see Table 1).

**Table 1:** Inclusion and exclusion criteria for the searches

	**CHC PRO concept search**	**CHC trials with PRO endpoints search**	**PRO measure search**
**Search Specific Inclusion Criteria**	CHC and over 16 years of age	CHC and over 16 years of age	CHC and over 16 years of age
**Search Specific Inclusion Criteria**	1999-2009	1994-2009	1999-2009
**Search Specific Inclusion Criteria**	Review article, qualitative research or article discussing concepts relevant to CHC disease or treatment.	· RCTs or non-randomized trials.	· Reported on the development/ validation of a PRO instrument or one of the concepts of interest selected during the CHC PRO Concept Search.
		· Patients in at least one arm must have received pegylated interferon.	
		· Follow-up duration of at least 48 weeks.	· PRO instrument designed for use in CHC population or used in CHC population; if no PROs available for a particular concept of interest additional methods were utilized.
		· PRO data must have been reported in a manner that allowed for determination of differentiation between treatments over time.	

CHC = Chronic Hepatitis C; RCT = Randomized controlled trials.

### Databases

Literature searches were conducted in PubMed and EMBASE databases. The CHC Trials with PRO Endpoints Search also included review of CHC clinical trials listed on the clinialtrials.gov website (1994–2009) and the PRO Measure Search included review of the Patient-Reported Outcome and Quality of Life Instruments Database (PROQOLID). All sources are widely used and accepted databases within the field of PRO measurement.

### Key search terms and screening process

Search terms for the CHC PRO Concept Search included the following terms: CHC, HCV, hep C, hepatitis C, chronic hepatitis C, and methodological terms such as qualitative, patient perspective, and focus group. Abstracts identified through PubMed and EMBASE were reviewed for eligibility, and eligibility was confirmed when reviewing full-text articles. All CHC concepts reported in the articles from the CHC PRO Concept Search were recorded, and a list of the most common concepts was generated.

Search terms for the CHC Trials with PRO Endpoints Search included the CHC terminology used in the CHC PRO Concept Search and terms for peginterferon alpha *2a* and clinical trials. Additionally, when possible this search was limited to clinical trial, controlled clinical trial, randomized controlled trial, or Phase I-IV clinical trials. Publications from relevant trials identified through a search of clinicaltrial.gov were reviewed for eligibility, and PRO measures were reviewed for inclusion in the CHC Trials with PRO Endpoints Search and the PRO Measure Search.

Results from the CHC PRO Concept Search and the CHC Trials with PRO Endpoints Search guided the PRO Measure Search, supplemented by findings from PubMed, EMBASE, PROQOLID, and previous research completed in CHC by the authors. From these multiple sources, a pool of potential PRO measures was generated for consideration.

## Results

### Findings from PRO concept search

**Figure 1:**
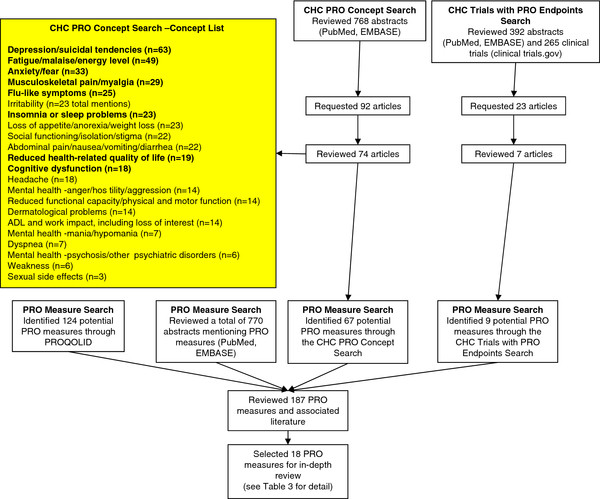
Literature search results summary for the searches.

Seven-hundred and sixty-eight (768) abstracts were reviewed during the PRO Concept Search. Ninety-two (92) of these articles underwent full-text evaluation, with 74 articles found to be relevant to the search objectives (Figure 1). These 74 articles included 31 review articles, 22 qualitative research articles that provided insight into the CHC patient perspective, and 23 observational or prospective study articles.

The PRO Concept Search identified 22 CHC-related concepts mentioned in three or more articles (Figure 1). Concepts included symptoms and treatment effects related to mental health, physical health, pain, HRQL, activities of daily living (ADL), cognitive dysfunction, and other categories. The most frequently reported CHC-related concepts were: depression [5,6,10,14-30], fatigue [5,6,10,14-26], anxiety/fear [5,6,10,14,17,18,21,22,25,27,28,30,31], musculoskeletal pain/myalgia [6,10,16-18,20,22-24,26], flu-like symptoms [6,10,17,18,23,24,26], irritability [6,16,18,23,29], insomnia or sleep problems [5,6,10,15,18-20,23,24], loss of appetite/anorexia/weight loss [6,10,17,20,23,24], social functioning/isolation/stigma [5,6,10,14-19,22,26-30], gastrointestinal symptoms [5,6,10,17,18,20,24,26], reduced HRQL [5,6,10,14,18,24,32], cognitive impairment [5,10,15,16,20,23,24], and headache [10,18,20,22,24].

Twenty-two of the articles reported on qualitative studies of CHC patients that provided valuable insight into the patient perspective [5,6,10,14-32]. The qualitative research reviewed identified similar concepts as those listed above. The most commonly reported symptoms were: fatigue [5,6,10,14-26], depression [5,6,10,14-30], anxiety/fear, and inability to function or social consequences of disease [5,6,10,14,17,18,21,22,25,27,28,30]. Other outcomes, including HCV symptoms and side effects of treatment, were: cognitive dysfunction [5,10,15,16,20,23,24], musculoskeletal pain [6,10,16-18,20,22-24,26], gastrointestinal symptoms [5,6,10,17,18,20,24,26], flu-like symptoms [6,10,17,18,23,24,26], dyspnea [18], headache [10,18,20,22,24], reduced functional capacity [6,10,15,18,22,24], reduced quality of life [5,6,10,14,18,24,32], insomnia or sleep problems [5,6,10,15,18-20,23,24], reduced sexual desire [24], loss of appetite, weight loss or anorexia [6,10,17,20,23,24].

The qualitative research articles also demonstrated that patients emphasized the impact of their CHC symptoms and medication side effects on ADL and HRQL. The most frequently mentioned impact was on social function [5,6,10,14-19,22,26-31], including reduction in social activities, loss of social support, erosion of social roles, and HCV-related stigma. Psychological consequences were discussed along with a range of emotions, including grief [17], guilt [6], frustration [21], shame [6,27], shock [17,22], denial [6,22], despair [27], embarrassment [26], irritability [6,16,18,23,29], and hostility or anger [6,10,15,17,21-23,25,29]. Other emergent themes included difficulty at work [5,6,10,15,18,24,26], uncertainty or a lack of knowledge about the virus [5,6,10,14,15,17,21,22,28,32], issues related to stigma [5,6,14,15,19,22,27,29,30], and discussion of treatment costs and benefits [6,10,14,18,19,21,23,25,26,28,29,32].

The overall list of concepts was evaluated based on ease of implementation into a clinical trial, potential to detect change over time in a clinical trial, how frequently the concepts appeared in the literature, how often the concepts were a primary focus of an article, and whether the concepts were independent or part of a larger group of concepts. The following five concepts were identified as being important to the CHC patients, easily implemented in a clinical trial and sensitive to change over time: 1) depression/anxiety; 2) fatigue; 3) flu-like symptoms; 4) cognitive function; and 5) insomnia.

All of these concepts were endorsed by patients as being bothersome symptoms or treatment effects related to CHC in the qualitative research studies [5,6,10,14-30]. The concept of flu-like symptoms was considered to be a probable result of the current SOC and a symptom that may be malleable to change especially as new therapies are developed. The concepts of depression and anxiety were combined because these concepts often overlap and there are existing single measures that cover both concepts (e.g., Hospital Anxiety and Depression Scale); thus the concepts were combined.

### PRO endpoint results from CHC trials

Three-hundred and ninety-two (392) abstracts were identified through EMBASE and PubMed (Figure 1). The clinicaltrials.gov search resulted in 265 clinical trials (Figure 1). Twenty-nine (29) of these trials reported including at least one PRO measure. After reviewing the clinical trial abstracts, 23 articles were retrieved, and seven of these publications met eligibility requirements (see Tables 2 and 3) [3,33-36]. Five of these reported outcomes based on the SF-36 Health Survey [3,33-36], and an additional trial reported outcomes based on the Hepatitis Quality of Life Questionnaire (HQLQ), which integrates the SF-36 with hepatitis-specific questions [37]. Four utilized the Fatigue Severity Scale (FSS) [3,33,34,36]. One trial each utilized the Work Productivity and Activity Impairment instrument (WPAI) [37], the Hamilton Depression Rating Scale (HAM-D) [38] and, the Zung Self-Rating Depression Scale (ZSRDS) [38] (see Table 3). Overall, examination of the PRO measures used in clinical trials for SOC for CHC demonstrated inadequate coverage of the concepts discussed above.

**Table 2:** **CHC Trials with PRO Endpoints Search** - **Clinical Trial Characteristics**

**Citation**	**Study Design and Treatment**	**Study Population**	**PRO Instruments**	**PRO Domains Assessed**	**PRO results**
Arora et al [33]	- Randomized, open-label, parallel group, controlled trial	CHC patients	- SF-36	-HRQL	Patients with an SVR had better SF-36 and FSS scores than both virological non-responders and patients in the untreated control group at the end of follow-up (week 72; Table 2). In patients who received combination therapy (groups A and B combined), the differences in mean HRQL scores between patients with an SVR and virological non-responders were statistically significant for five of eight SF-36 domains (General Health, P < 0.0001; Bodily Pain Index, P < 0.0001; Role Physical, P < 0.05; Physical Functioning, P < 0.05; Vitality, P < 0.0001) and the Physical Health component score, P < 0.0001 (Table 2). Consistent with these findings, both the FSS Total score (P < 0.01 vs. non-responders) and VAS score (P < 0.01 vs. non-responders) were significantly better in patients with an SVR.
		N = 491	- FSS	-Impact and severity of fatigue on HRQL	
	- Group A = 24 weeks;	Prior therapy: Patients had not received prior treatment for CHC.			
	Group B = 48 weeks;				
	No treatment = 72 weeks				
	-Pegylated interferon alpha 2a + ribavarin				
Bernstein et al. [3]	- Open-label RCT	CHC patients	- SF-36	-Physical function, role limitations physical, vitality, general health perceptions, pain, social function, role limitations-emotional, mental health.	(Results using ANCOVA)
				-Fatigue	
	- 72 weeks	N = 676	- FSS		'Virologic response associated with positive HRQL changes in all domains on FSS and SF-36
	-Pegylated interferon alpha-2a; unmodified interferon-2a				
		Prior therapy: none			- Patients taking peginterferon reported better HRQOL and less fatigue than interferon in all FSS domains, and 7/8 of SF-36 domains (exception Physical Function).
Hassanein et al [34]	- Open-label RCT	CHC patients	- SF-36	-Physical function, role limitations physical, vitality, general health perceptions, pain, social function, role limitations-emotional, mental health	(Results using ANCOVA)
	- 72 weeks	N = 1121	- FSS		- Using the SF-36, the addition of ribavarin to peginterferon increased HRQOL in domains of physical function, role limitations, vitality, social functioning, mental health (not bodily pain, general health, role limitations emotional).
	-Peginterferon alfa-2a +placebo; Peginterferon alfa-2a + ribavarin; Interferon alfa2b + ribavarin				
		Prior therapy: None			
					- Using the FSS, the addition of ribavarin to peginterferon increased HRQOL in domains of total fatigue and fatigue severity.
				-Fatigue	
					- Using the SF36, peginterferon (with ribavarin) as compared to interferon (with ribavarin) increased HRQOL in domains of role limitations physical, bodily pain, vitality, social functioning (not physical function, general health, role limitations emotional, and mental health).
					- Using the FSS, peginterferon (with ribavarin) as compared to interferon (with ribavarin) increased HRQL in domains total fatigue and fatigue severity.
Nayman Alpat et al. [35]	- Non-randomized trial	CHC patients	- SF-36	-Physical function, role limitations physical, vitality, general health perceptions, pain, social function, role limitations-emotional, mental health	Use of the Mann Whitney U test revealed no significant differences between the treatment groups on any of the domains.
		N = 40			
	- 72 weeks	Prior therapy: Not Reported			
	-Peginterferon alfa-2a +ribavirin; Peginterferon alfa-2b +ribavirin				
Neri et al. [38]	- Randomized, open-label, prospective trial	HCV patients	- HAM-D	Depression	Logistic regression analysis showed in both groups a significant trend from baseline according to the ZSRDS (odds ratio 0.698, p < 0.01 [95% CI 0.59, 0.78]) to predict early major depressive disorders confirmed by the study.
		N = 186	- ZSRDS		
		Prior therapy: Not Reported			
	- Follow-up once a month for 48 weeks of treatment and 4 and 8 weeks after the end of therapy		- SCID		
	-Pegylated interferon alpha 2a; Pegylated interferon alpha 2b				
Perrillo et al. [37]	- Open-label RCT	CHC patients	- HQLQ	- HQLQ: social function, role limitations emotional, vitality, general mental health, physical function, role limitations physical, freedom from pain, general health, health distress, positive well-being, hepatitis-specific limitations, hepatitis-specific health, PCS, MCS.	ANCOVA was used to test differences in change scores between treatment groups.
	- 72 weeks	N = 412	- WPAI		
	-Peginterferon alfa-2&z.ausco;; Interferon alfa-2b + ribavirin	Prior therapy: None			
					- HRQL as measured on all domains diminished during treatment for both treatment groups.
					- For all SF-36 scales, however, the peginterferon alfa-2a group experienced less impairment than did the interferon alfa-2 b group.
					- The between-treatment differences were significant in 3 of the scales at week 48.
				-WPAI: impact of therapies on work productivity, health care utilization	
Rasenack et al. [36]	- Open-label, randomized, parallel-dose study	CHC patients	- FSS	- HRQL	-FSS- Statistically significant differences between treatment groups favoring peginterferon α-2a were seen in FSS total scores at weeks 2, 12, and 24 and in FSS VAS scores at weeks 2 and 12 (p < 0.01).
		N = 531	- SF-36	- Fatigue	
		Prior therapy: None			
	- 72 weeks				
					-SF-36- peginterferon α-2a patients demonstrated significantly better mean scores in all eight SF-36 domains (p < 0.01 to p < 0.05) compared with those treated with unmodified interferon α-2a.
	-Peginterferon α-2a (40kD); Unmodified interferon α-2a				

FSS - Fatigue Severity Scale; HAM-D - Hamilton Rating Scale for Depression; HQLQ - Hepatitis Quality of Life Questionnaire; SCID - structured clinical interview for DSM-IV disorders; SF-36 - Short-form Health Survey; WPAI - Work Productivity and Activity Impairment; ZSDRS - Zung Self-Rating Depression Scale.

**Table 3:** PRO measures identified by concept of interest

**Depression and anxiety**	**Fatigue**	**Flu**-**like symptoms**	**Cognitive function**	**Insomnia**	**CHC**-**specific measures**
BDI and revised BDI-II	FIS	Flu-iiQ	MOS-Cog	ESS	CLDQ^1^
BAI	FSS^1^	FIQ	MOS-SS	PSQI	CLDQ-HCV^1^
HADS	MAF			PROMIS Sleep Disturbance Subscale	HQLQ^1^
CES-D	PROMIS Fatigue Subscale				
ZSRDS					
PROMIS Depression Subscale					

**Depression and anxiety**: BDI - Beck Depression Inventory; BAI - Beck Anxiety Inventory questionnaire; HADS - Hospital Anxiety and Depression Scale; CES-D - Center for Epidemiologic Studies - Depression Scale; ZSRDS - Zung Self-Rated Depression Scale; PROMIS - Patient Reported Outcome Measurement Information System. **Fatigue**: FIS - Fatigue Impact Scale; FSS - Fatigue Severity Scale; MAF - Multidimensional Assessment of Fatigue; PROMIS - Patient Reported Outcome Measurement Information System. **Flu**-**like symptoms**: Flu-iiQ - Symptom Intensity and Impact of Influenza Questionnaire; FIQ - Fibromyalgia Impact Questionnaire. Note the Flu-iiQ was previously two measures, the Influenza Symptom Severity scale (ISS) and the Influenza Impact Wellbeing Scale (IIWS). The Flu-iiQ is a newer version of the measures that encompasses both the ISS and IIWS. **Cognitive Function**: MOS-Cog - Medical Outcomes Study Cognitive Scale; MOS-SS - Medical Outcomes Study Sleep Scale. **Insomnia**: ESS - Epworth Sleepiness Scale; PSQI - Pittsburgh Sleep Quality Index; PROMIS - Patient Reported Outcome Measurement Information System Sleep Disturbance subscale. **CHC specific measures**: CLDQ - Chronic Liver Disease Questionnaire; CLDQ-HCV - Chronic Liver Disease Questionnaire-Hepatitis C Virus; HQLQ - Hepatitis Quality of Life Scale/Hepatitis Quality of Life Questionnaire.

^1^ Four of the measures reviewed were validated in CHC populations, the FSS, CLDQ, CLDQ-HCV, and HQLQ.

### Review of PRO measures

Eighteen PRO measures were selected for in-depth review based on the findings of the three searches and the a priori eligibility criteria (Table 1). Table 3 lists the 18 PRO measures by concept of interest. Instrument characteristics such as the population in which the instrument was developed or validated, the methods of instrument development, scale and scoring information, recall period, and mode of administration were reviewed. Four of the 18 PRO measures selected were validated in CHC populations: Fatigue Severity Scale (FSS), the Chronic Liver Disease Questionnaire (CLDQ), the Chronic Liver Disease Questionnaire-Hepatitis C Virus (CLDQ-HCV), the Hepatitis Quality of Life Questionnaire (HQLQ) [39,40]. Instrument development and psychometric validation articles were examined for these four PRO measures. Each of these measures has demonstrated acceptable psychometric characteristics, including internal consistency reliability [41,42], test-retest reliability [41], construct validity [39-42], and known-groups validity [39,41,42].

## Conclusion and discussion

Chronic hepatitis C and its treatment are associated with a wide variety of symptoms and impacts as reported by patients. We examined a variety of published CHC studies in order to identify concepts that capture the patient experience of CHC and have the potential to be sensitive to change due to treatment. Based on these considerations, five concepts of interest were selected for further investigation within publications of clinical trials with CHC patients that included PRO measurement and publications of PRO development and psychometric evaluation studies. These five concepts were selected based on their perceived sensitivity to change in disease status, either by progression or treatment, and sensitive to treatment effects. Our goal was to determine if the concepts we identified through the literature as providing insight into the perspective of CHC patients had been translated into PRO instruments, and whether the published clinical trials reported PRO data that reflect clinical changes, ideally within the concepts that were identified as important to CHC patients.

Examination of the PRO measures used in clinical trials for SOC for CHC demonstrated inadequate coverage of the concepts of: 1) depression/anxiety; 2) fatigue; 3) flu-like symptoms; 4) cognitive function; and 5) insomnia. Instruments measuring HRQL were frequently included in clinical trials. Clinical trials also sometimes included measures of fatigue, depression, and anxiety, but were less likely to include measures of cognitive function, insomnia, and flu-like symptoms. Incorporating PRO measures of the concepts of interest that have been validated for use within CHC populations into clinical trials would provide additional useful information on compliance and treatment maintenance. Further, instruments are available to measure almost all of the top five concepts identified as being important to patients; that is, depression/anxiety, fatigue, flu-like symptoms, cognitive function, and insomnia.

Qualitative studies evaluating the content validity of PRO measures are important to verify whether or not the instrument is measuring concepts that are relevant and important to patients. Only one of the 18 instruments reviewed, the HQLQ, had evidence demonstrating content validity in the CHC population. The FSS and CLDQ, both psychometrically validated in the CHC population, included qualitative interviews with patients as part of the PRO development process, but the qualitative research was not specifically conducted in the CHC population.

### Recommendations for measuring the concept of depression/anxiety

The Hospital Anxiety and Depression Scale (HADS) may be a potential PRO instrument for use in measuring anxiety and depression in CHC studies. The advantages of the HADS is that it is a single, short questionnaire and has been used in prior clinical trials of CHC as well as with many other physical illnesses to measure the psychological impact of both disease and treatment. The HADS is a well-validated measure in other disease areas; however, it is lacking any type of content validity or psychometric evidence in a CHC sample. Qualitative research is recommended to assess whether the HADS anxiety and depression items cover relevant aspects of the CHC experience and to ensure that CHC patients understand the items, response scales, and instructions. In addition, studies aimed at evaluating the reliability and validity of the HADS within the CHC population are needed.

### Recommendations for measuring the concept of fatigue

The Fatigue Severity Scale (FSS) may be a useful PRO measure for assessing fatigue-related symptoms in CHC. The FSS covers fatigue-related symptoms and impact of fatigue on functioning. The FSS has been used in several prior trials comparing treatments for CHC and has been demonstrated to be responsive to change over time and predictive of treatment discontinuation. Additionally, it is a short questionnaire, which may result in little burden on patients. The FSS has demonstrated excellent psychometric characteristics in the CHC [41]. Qualitative research on the FSS within CHC populations is needed to determine if the FSS item content is consistent with the patient experience in CHC.

### Recommendations for measuring the concept of flu-like symptoms

Several instruments are available to measure influenza symptoms; however, not all symptoms of influenza are relevant to the general malaise often felt by patients with CHC. Symptoms such as nasal congestion, sore throat, and cough are more unique to influenza and would not be appropriate for use in CHC. Future qualitative research is needed to identify which flu-like symptoms are most salient and relevant to patients with CHC. The goal of this research would be to develop a daily diary or PRO measure that can be used to assess the most common flu-like symptoms.

### Recommendations for measuring the concept of cognitive function

There are many existing neuropsychological tests and batteries that are available to measure all aspects of cognitive function. Administration of neuropsychological tests and batteries requires training for individuals who will administer the test, which can be time-consuming and have cost implications. Further neuropsychological tests may not accurately reflect patients’ complaints and may not be able to measure impairment levels that are relevant for CHC. One alternative to neuropsychological tests and batteries may be to utilize a PRO measure, such as the Medical Outcomes Study – Cognitive Scale (MOS-Cog), to assess cognition [43,44]. This measure contains six items that cover reasoning, concentration and thinking, confusion, memory, attention, and psychomotor function. Currently, the MOS-Cog is not validated in CHC populations; however, it was used in general primary care surveys, such as the Medical Outcomes Study [44]. Qualitative and quantitative research is needed before the MOS-Cog can be confidently used in CHC studies.

### Recommendations for utilizing item banks

There are also recently developed measures of depression, anxiety, fatigue, and sleep disturbance, based on item banks, from the National Institutes of Health PROMIS project [45-48], that have the potential to improve measurement of these PRO domains. Item banks enable intelligent design of short-form scales where items are selected to best assess the construct based on knowledge about the patient’s health status. In addition, these item banks allow for computer adaptive testing, which involves tailoring the measure to individual patients. Computer adaptive tests are individualized and can be set for specific levels of measurement precision (i.e., reliability) at the individual level. These types of applications and methods may represent the future of health outcomes assessment.

### Limitations

Several limitations should be considered related to this review. First, this review only focused on a limited number of CHC-related concepts. These concepts, although relevant to patients with CHC, do not encompass all the PRO domains relevant to CHC. This was primarily because of our focus on providing recommendations for PRO measures to be used in CHC clinical trials. Second, we limited the review to SOC clinical trials to identify instruments measuring the previously identified concepts. Therefore, some PRO concepts included in earlier clinical trials may have been missed. Finally, we did not examine available PRO instruments developed using a mixed population of patients with a variety of liver diseases [49-51] but focused solely on PROs developed with patient populations that were solely CHC or PRO measures developed for concepts identified that were of interest to CHC patients. Instruments developed using mixed patient populations pooled the results and did not report solely on the CHC patients.

### Summary

In summary, this review identified gaps in the PRO measurement literature regarding assessment of symptoms and outcomes that are reported to be important by patients with CHC. Further research is needed to ensure that the measures used in CHC clinical trials are measuring concepts that were identified as important to CHC patients and have evidence supporting content validity, reliability, construct validity, and responsiveness. Some qualitative research has been completed in CHC patients, and the information from these studies can then be used to develop new measures to assess relevant PRO domains. Clearly, PROs are necessary for assessing the impact of CHC and treatment for CHC from the patient’s perspective, and these PRO endpoints are important to include in CHC clinical trials.

## Abbreviations

ADL: Activities of daily living; CHC: Chronic hepatitis C; CLDQ: Chronic Liver Disease Questionnaire; CLDQ-HCV: Chronic Liver Disease Questionnaire-Hepatitis C Virus; FDA: Food and Drug Administration; FSS: Fatigue Severity Scale; HADS: Hospital Anxiety and Depression Scale; HAM-D: Hamilton Depression Rating Scale; HCV: Hepatitis C virus; HQLQ: Hepatitis Quality of Life Questionnaire; IFN-α: Interferon alpha; HRQL: Health-related quality of life; MOS-Cog: Medical Outcomes Study – Cognitive Scale; PRO: Patient-reported outcome; PROQOLID: Patient-Reported Outcome and Quality of Life Instruments Database; RBV: Ribavirin; SOC: Standard of care; WPAI: Work Productivity and Activity Impairment; ZSRDS: Zung Self-Rating Depression Scale.

## Competing interests

This manuscript was supported by an unrestricted grant from Bristol-Myers Squibb. YY and GL are employed by Bristol-Myers Squibb. LK, SM, SK, and DR are employed by United BioSource Corporation, who received support to complete this manuscript.

## Authors’ contributions

LK served as the Principal Investigator, overseeing the direction of the project and reviewing all results. SK and SM participated in the implementation of the project, designing the literature search; reviewing the results, publications, and measures; and synthesizing the results. LK, SM, YY, DR, and GL participated in the design of the study and performed the statistical analysis. YY and GL conceived of the study, participated in its design and coordination, and helped to draft the manuscript. All authors read and approved the final manuscript.
